# Detection of Salivary miRNAs That Predict Chronic Periodontitis Progression: A Cohort Study

**DOI:** 10.3390/ijerph18158010

**Published:** 2021-07-29

**Authors:** Kohei Fujimori, Toshiki Yoneda, Takaaki Tomofuji, Daisuke Ekuni, Tetsuji Azuma, Takayuki Maruyama, Yoshio Sugiura, Manabu Morita

**Affiliations:** 1 Department of Preventive Dentistry, Graduate School of Medicine, Dentistry and Pharmaceutical Sciences, Okayama University, 2-5-1 Shikata-cho, Kita-ku, Okayama 700-8558, Japan; kfujimori@s.okayama-u.ac.jp (K.F.); de17057@s.okadai.jp (T.Y.); dekuni7@md.okayama-u.ac.jp (D.E.); t-maru@md.okayama-u.ac.jp (T.M.); de421022@s.okayama-u.ac.jp (Y.S.); 2 Department of Community Oral Health, School of Dentistry, Asahi University, 1851-1 Hozumi, Mizuho 501-0296, Japan; tomofu@dent.asahi-u.ac.jp (T.T.); tetsuji@dent.asahi-u.ac.jp (T.A.); 3 Advanced Research Center for Oral and Craniofacial Sciences, Dental School, Okayama University, 2-5-1 Shikata-cho, Kita-ku, Okayama 700-8558, Japan

**Keywords:** saliva, periodontitis, microRNAs, precision medicine, cohort studies

## Abstract

The aim of this two-year cohort study was to investigate salivary microRNAs (miRNAs) that predict periodontitis progression. A total of 120 patients who underwent supportive periodontal therapy were recruited. Unstimulated whole saliva was collected at baseline. Two years later, 44 patients were followed up (median age, 67.1 years) and divided into two groups: progression group (*n* = 22), with one or more sites with clinical attachment level (CAL) progression (>3 mm compared with baseline) or tooth extraction due to periodontitis progression; and the control group (*n* = 22), which did not exhibit CAL progression. In the microarray analysis of salivary miRNAs, hsa-miR-5571-5p, hsa-miR-17-3p, hsa-let-7f-5p, hsa-miR-4724-3p, hsa-miR-99a-5p, hsa-miR-200a-3p, hsa-miR-28-5p, hsa-miR-320d, and hsa-miR-31-5p showed fold change values <0.5 or ≥2.0 in the progression group compared with the control group (*p* < 0.05). On receiver operating characteristic curve analysis, areas under the curves of hsa-miR-5571-5p, hsa-let-7f-5p, hsa-miR-99a-5p, hsa-miR-28-5p, and hsa-miR-320d were >0.7, indicating fair discrimination power. The expressions of salivary hsa-miR-5571-5p, hsa-let-7f-5p, hsa-miR-99a-5p, hsa-miR-28-5p, and hsa-miR-320d were associated with periodontitis progression in patients with chronic periodontitis. These salivary miRNAs may be new biomarkers for progression of periodontitis, and monitoring them may contribute to new diagnostics and precision medicine for periodontitis.

## 1. Introduction

Recently, the concept of precision medicine has attracted attention [1]. Precision medicine is a novel approach that predicts or diagnoses the disease before onset. In particular, precision medicine comprises “treatments targeted to the needs of individual patients on the basis of genetic, biomarker, phenotypic or psychosocial characteristics that distinguish a given patient from other patients with similar clinical presentations” [2].

One of the candidate biomarkers for precision medicine is microRNAs (miRNAs), which are short sequences, generally 19–25 nucleotides, that regulate post-transcriptional silencing of targeted mRNAs, blocking translation or inducing degradation of mRNA [3]. miRNAs are implicated in several physiological and pathological mechanisms and play important roles in inflammatory responses and the development of diseases including cancer and rheumatoid arthritis [4]. miRNAs can be biomarkers for cancer diagnosis and prognosis [5], and specific serum miRNAs have been used as diagnostic tools for malignancies, autoimmune diseases, and infectious diseases [6]. For example, a systematic review suggested moderate diagnostic accuracy of the blood and salivary miRNAs presented for oral squamous cell carcinoma, including miR-21 and miR-233 [7]. The expression of serum exosomal miR-301a serves as a useful biomarker for glioma [8].

Saliva has been studied thoroughly as a potential diagnostic tool because its collection is non-invasive and economical. Salivary miRNA expression is modulated by various systemic diseases [9,10]. The expression of salivary miR-4484 is a potential biomarker in oral lichen planus [11]. Furthermore, salivary miR-455-3p is useful as a complementary tool in the diagnosis of adenoid cystic carcinoma [12].

Periodontitis is one of the most complex non-communicable diseases. Periodontitis progression results in periodontal attachment loss and/or tooth loss. To prevent periodontitis progression, realization of precision medicine for periodontitis is desired. Measuring salivary mediators—such as lactate dehydrogenase, lactoferrin, and 8-hydroxydeoxyguanosine—could be useful in diagnosing the periodontal condition [13,14]. Previous studies have reported that periodontal disease alters miRNA expression in human periodontal tissues [15,16,17]. Recently, specific serum and gingival crevicular fluid miRNAs were identified as biomarkers for chronic periodontitis [18,19]. However, the details remain unclear because these studies were cross-sectional, not longitudinal. A recent review suggested that a few salivary miRNAs have high diagnostic, prognostic, and therapeutic potential [20]. Nevertheless, it also suggested that further investigations are required, because there are not enough diagnostic and prognostic biomarkers of periodontal disease. Thus, we hypothesized that salivary miRNAs can be useful as biomarkers for predicting periodontitis progression. The aim of this cohort study was to identify miRNAs associated with periodontitis progression among chronic periodontitis patients receiving supportive periodontal therapy, which will contribute to predicting the onset and progression of periodontitis and achieving precision medicine in the field of oral health.

## 2. Materials and Methods

### 2.1. Experimental Design

This two-year, prospective, cohort study was performed from 2016 to 2018. This study was conducted in accordance with the Declaration of Helsinki and approved by the Ethics Committee of Okayama University Graduate School of Medicine, Dentistry and Pharmaceutical Sciences and Okayama University Hospital (No. 1603-002). The study was conducted at the Department of Preventive Dentistry, Okayama University Hospital in Japan. At baseline, patients were recruited from July 2016 to October 2016.

### 2.2. Participants

At baseline, patients who visited the Department of Preventive Dentistry, Okayama University Hospital, every 3 to 6 months for supportive periodontal therapy were recruited. Inclusion criteria were patients who were originally recruited in our pilot study [21] and provided written, informed consent. All participants were Japanese adult men and women over 40 years of age. The participants did not have acute symptoms, oral pain, oral disorders, or dental treatments other than supportive periodontal therapy. The periodontal condition was stable in all patients. The frequency of supportive therapy was determined based on the condition of chronic periodontitis and the self-care ability of patients. There was no difference in frequency between the two groups. Exclusion criteria were as follows: patients with <20 natural teeth, smoking habit, anti-inflammatory drug use, samples with low-quality RNA, or dropped out.

Before oral examination, whole saliva samples were collected at baseline. Then, a cohort of patients was followed-up for up to two years of supportive periodontal therapy. During the study period, the periodontal condition was evaluated at each visit. At the end of the study period, the patients were divided into two groups. The progression group was defined as those who had one or more sites with clinical attachment level (CAL) progression (>3 mm compared with baseline) or tooth extraction due to periodontitis progression during the two-year study period [22]. The control group was defined as patients without CAL progression.

### 2.3. Oral Examination

Dentists examined probing pocket depth (PPD), CAL, and bleeding on probing (BOP) at baseline and each reassessment. PPD and CAL at six sites of all teeth (mesio-buccal, mid-buccal, disto-buccal, mesio-lingual, mid-lingual, and disto-lingual) were measured using a periodontal probe (Hu-Friedy, Chicago, IL, USA). The percentage of bleeding sites after probing (%BOP) was calculated. Dental plaque was disclosed with erythrosine. O’Leary’s plaque control record was used for evaluation of oral hygiene [23]. Five trained and calibrated dentists (D.E., T.A., T.M., T.T. and T.Y.) carried out oral examinations. Kappa coefficients for intra- and inter-examiner and intra-class correlation coefficients were >0.8.

### 2.4. Questionnaire

Participants answered a questionnaire about drug history, systemic diseases, and smoking status [24].

### 2.5. Saliva Collection and RNA Extraction

Dentists collected unstimulated whole saliva (1–2 mL) at baseline from 07:00 to 12:00 to exclude the effects of circadian rhythm, just before oral examination [25]. Samples were centrifuged for 10 min at 2000× *g* at room temperature, and then supernatants were collected and stored at −80 °C until use.

Exosomes were extracted from samples using Total Exosome Isolation Reagent (Invitrogen, Carlsbad, CA, USA). Next, total RNA was isolated using total exosome RNA and protein isolation kits (Invitrogen) [26]. The quality of total RNA was determined using Agilent 2100 Bioanalyzer (Agilent Technologies, Santa Clara, CA, USA).

### 2.6. miRNA Analysis

The miRNA analysis was conducted in four phases: (i) screening phase, (ii) selection phase, (iii) validation phase, and (iv) bioinformatics phase. In the screening phase, pooled RNA samples (*n* = 3) from the progression group or the control group (*n* = 3) were prepared separately using 3D-Gene RNA extraction reagent (Toray, Kanagawa, Japan). Samples were labeled with the 3D-Gene miRNA labeling kit (Toray, Kanagawa, Japan). Then, samples were hybridized to the microarray chip (3D-Gene Human miRNA Oligo chip v20) according to the manufacturer′s instructions. Annotation and sequences of oligonucleotide probes were based on miRBase 22 (the miRNA database) (http://www.mirbase.org/ (accessed on 11 October 2019)). The 3D-Gene Scanner (Toray, Kanagawa, Japan) was used for analysis of fluorescent signals. Data were then assessed using 3D-Gene Extraction software (Toray, Kanagawa, Japan). Data were normalized by replacing background signals with the mean intensity. Signal intensities were calculated by subtracting the background signal from the dots with intensities >2 standard deviations compared with those of background. The median signal intensity was set as 25.0. The fold change in values of the progression group was calculated for each miRNA in reference to signals in the control group. In the selection phase, miRNAs showing significant fold changes (<0.5 or >2, *p* < 0.05) were chosen.

miRNAs were selected in the selection phase. Briefly, significant differentially expressed miRNAs with fold changes <0.5 or >2 (*p* < 0.05) in the progression group compared with the control group were selected.

In the validation phase, quantitative real-time polymerase chain reaction (RT-qPCR) analysis was performed on the Mx3000P Real-time QPCR System (Agilent Technologies) in triplicate for all samples. Analyses were performed using TaqMan microRNA Assays (Thermo Fisher Scientific, Waltham, MA) [27,28]. Cycle threshold (Ct) values were transformed using the 2-ΔCt method (ΔCt, Ct value of each gene minus the lowest Ct value of the corresponding gene in different samples) [25]. U6 snRNA was used as internal control miRNA. Receiver operating characteristic (ROC) curves were constructed by plotting 1 − specificity on the *X*-axis and the sensitivity on the *Y*-axis to show the association between the true-positive rate and the false-positive rate for selected cut-off values [29]. Then, areas under the curves (AUCs) were calculated to determine the predictive power of cut-off values, and the Youden index (sensitivity + specificity − 1) was used to select the optimal threshold value [30].

### 2.7. Bioinformatics

In the bioinformatics phase, targeted genes of miRNAs were confirmed using the system of miRWalk [31] to investigate the role and function of these miRNAs. Furthermore, several bioinformatics databases such as TargetScan, miRanda, and miRWalk were used, focusing on targets that were present in at least two databases. Signaling pathways of targeted genes were selected using GeneCodis 3.0 [32,33,34].

### 2.8. Statistical Analysis

Student’s *t*-test, the chi-squared test in the screening phase, analysis of covariance, or the Mann–Whitney *U*-test was used to determine the significance of differences between the progression and control groups. All statistical analyses were conducted using SPSS statistics version 22.0 J (IBM Japan, Tokyo, Japan), and *p* < 0.05 was considered significant.

## 3. Results

### 3.1. Patient Characteristics

**Figure 1:**
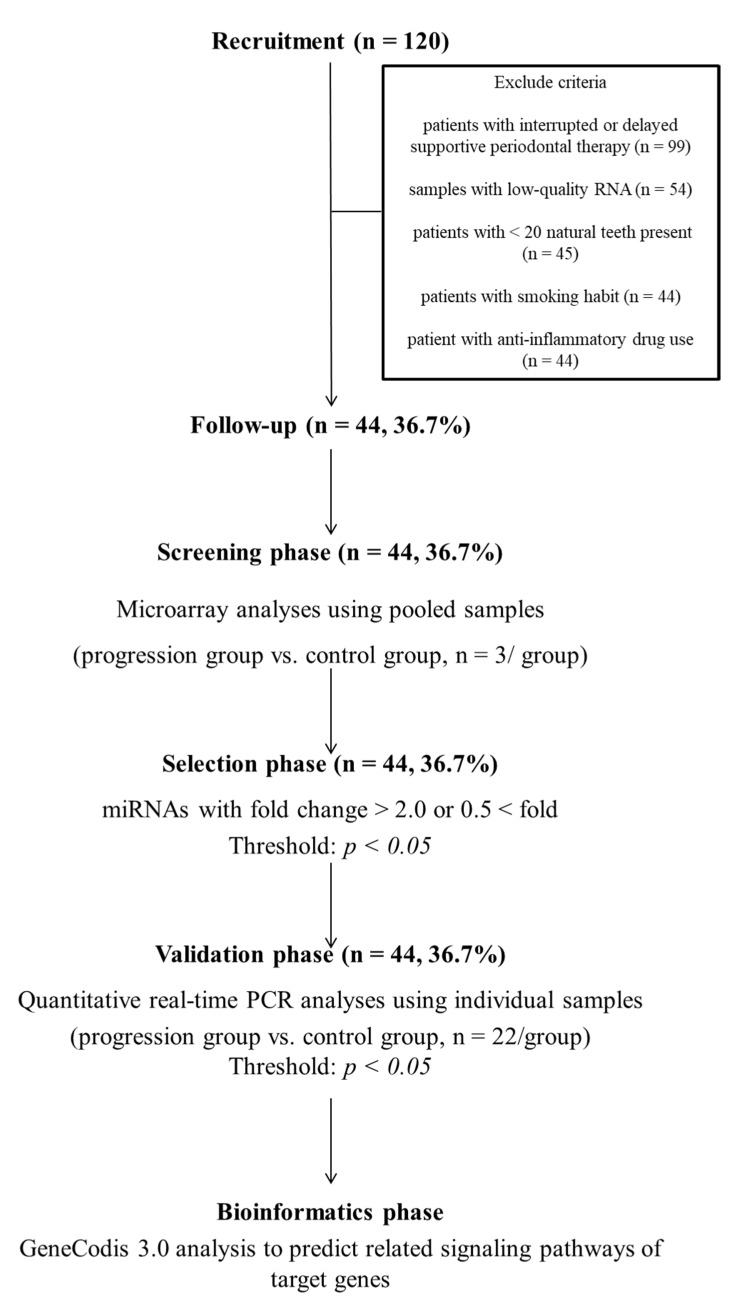
An overview of the experimental design.

Finally, complete data and quality samples were available for 44 of 120 patients initially enrolled in this study (Figure 1). These 44 participants were included in subsequent analyses. In the progression group, three patients underwent tooth extractions. However, there were no significant differences in characteristics between participants and non-participants (Table 1 and Table S1, respectively).

### 3.2. Microarray Analysis

There were significant differences in the expression levels of hsa-miR-5571-5p, hsa-miR-17-3p, hsa-let-7f-5p, hsa-miR-4724-3p, hsa-miR-99a-5p, hsa-miR-200a-3p, hsa-miR-28-5p, hsa-miR-320d, and hsa-miR-31-5p between the progression and control groups (*p* < 0.05) (Table 2).

### 3.3. Validation of miRNAs

**Figure 2:**
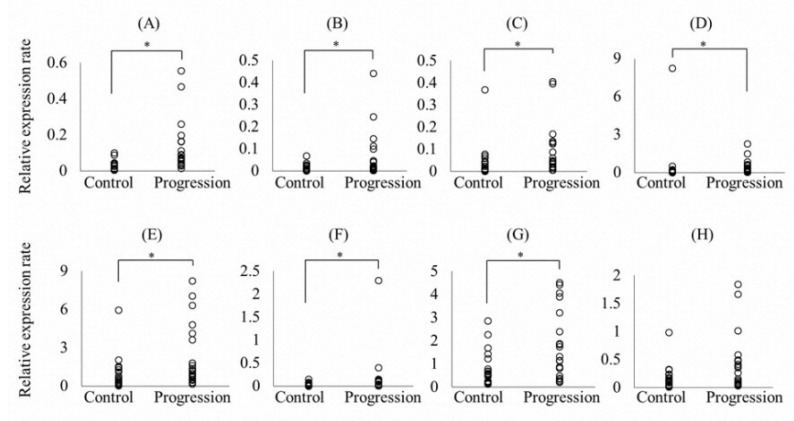
Relative expression rates of hsa-miR-5571-5p (**A**), hsa-miR-17-3p (**B**), hsa-let-7f-5p (**C**), hsa-miR-99a-5p (**D**), hsa-miR-200a-3p (**E**), hsa-miR-28-5p (**F**), hsa-miR-320d (**G**), and hsa-miR-31-5p (**H**) in saliva using quantitative real-time PCR assays. The relative expression rate of each miRNA was normalized to that of U6 snRNA. Each individual sample is shown as a circle (*n* = 22/group). * *p* < 0.05, Mann–Whitney *U*-test.

Because primers to detect hsa-miR-4724 are not commercially available, other miRNAs were selected for RT-qPCR assays. There were significant differences in the expressions of hsa-miR-5571-5p, hsa-miR-200a-3p, hsa-let-7f-5p, hsa-miR-17-3p, hsa-miR-99a-5p, hsa-miR-28-5p, and hsa-miR-320d between the progression and control groups (*p* < 0.05, Mann–Whitney *U*-test) (Figure 2).

### 3.4. Discrimination Power of hsa-miR-5571-5p, hsa-miR-17-3p, hsa-let-7f-5p, hsa-miR-99a-5p, hsa-miR-200a-3p, hsa-miR-28-5p, and hsa-miR-320d for the Progression Group

**Figure 3:**
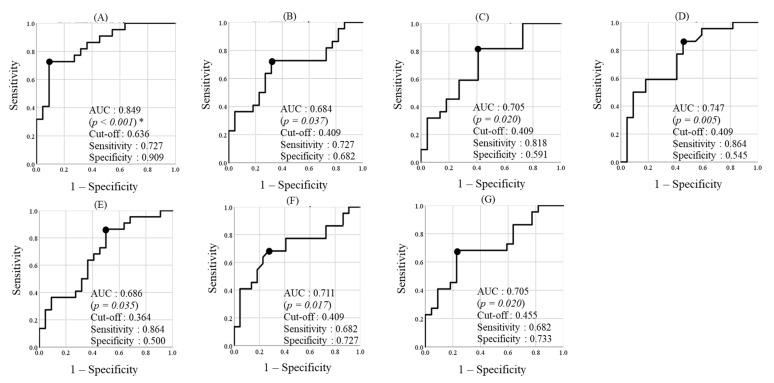
Receiver operating characteristic (ROC) curve analysis for the discriminatory power of miRNAs for the progression and control groups. ROC curves for hsa-miR-5571-5p (**A**), hsa-miR-17-3p (**B**), hsa-let-7f-5p (**C**), hsa-miR-99a-5p (**D**), hsa-miR-200a-3p (**E**), hsa-miR-28-5p (**F**), and hsa-miR-320d (**G**) are shown based on the relative expression ratio. Sensitivity, specificity, and cut-off values were optimized using the maximum Youden index. The solid line with the black circle shows the plot with these optimized values. * non-parametric test for the area under the curve (AUC).

The discrimination powers of hsa-miR-5571-5p, hsa-miR-17-3p, hsa-let-7f-5p, hsa-miR-99a-5p, hsa-miR-200a-3p, hsa-miR-28-5p, and hsa-miR-320d were next examined by ROC curve analysis (Figure 3). AUCs were 0.849 for hsa-miR-5571-5p (*p* < 0.001), 0.684 for hsa-miR-17-3p (*p* = 0.037), 0.705 for hsa-let-7f-5p (*p* = 0.02), 0.747 for hsa-miR-99a-5p (*p* = 0.0054), 0.686 for hsa-miR-200a-3p (*p* = 0.035), 0.711 for hsa-miR-28-5p (*p* = 0.017), and 0.705 for hsa-miR-320d (*p* = 0.02). AUCs of hsa-miR-5571-5p, hsa-let-7f-5p, hsa-miR-99a-5p, hsa-miR-28-5p, and hsa-miR-320d were >0.7.

### 3.5. Analysis of Covariance

In addition, analysis of covariance was used to calculate the unstandardized regression with 95% confidence interval (CI) for progression of periodontitis. The expression of hsa-miR-5571-5p was associated with progression of periodontitis, with an unstandardized regression coefficient-0.092 (95% CI: −0.162, −0.022, *p* = 0.011) (Table S2).

### 3.6. Bioinformatics

These miRNAs were further processed for pathway analyses. Results of pathway analyses demonstrated that hsa-miR-5571-5p, hsa-let-7f-5p, hsa-miR-200a-3p, hsa-miR-28-5p, and hsa-miR-320 were associated with MAPK signaling, whereas hsa-miR-99a-5p was associated with chemokines (Table S3).

## 4. Discussion

To the best of our knowledge, this is the first cohort study of the association between salivary miRNA expression and periodontitis progression. We hypothesized that saliva miRNAs correlated with periodontitis progression. In microarray analysis, nine miRNAs were selected as biomarkers to predict chronic periodontitis progression, and relative expressions of these miRNAs were confirmed by RT-qPCR. The expression of seven miRNAs was significantly higher in the progression group compared with the control group. These results showed that measuring the expression of these miRNAs could be useful to identify the progression group. On ROC curve analysis, AUCs of hsa-miR-5571-5p, hsa-let-7f-5p, hsa-miR-99a-5p, hsa-miR-28-5p, and hsa-miR-320d were >0.7, indicating fair discrimination power [35]. These microRNAs could be useful to predict periodontitis progression. Thus, these findings suggest that it is possible to predict the onset and progression of periodontitis and to achieve precision medicine in the field of oral health.

Bioinformatics analysis suggested that target genes of hsa-miR-5571-5p, hsa-let-7f-5p, hsa-miR-200a-3p, hsa-miR-28-5p, and hsa-miR-320 were involved in the pathology of periodontitis. These miRNAs are involved in the MAPK signaling pathway. The MAPK signaling pathway is related to apoptosis and inflammation. The MAPK signaling pathway is also associated with the pathology of periodontitis [36]. Thus, modulated expression of these miRNAs in saliva may affect periodontitis progression through the MAPK signaling pathway. In addition, hsa-miR-99a-5p was associated with chemokines. Chemokines mainly regulate leukocyte migration. Therefore, the high expression of hsa-miR-99a-5p may suppress leukocyte migration and affect periodontitis progression.

A study reported that hsa-let-7f-5p expression was upregulated in periodontal gingival tissues [15]. The present finding is consistent with the results of that study; thus, measuring hsa-let-7f-5p expression may be useful to predict periodontitis in precision medicine. Other miRNAs were not reported in similar studies about the association between miRNAs and periodontitis. Therefore, the present findings of these miRNAs may indicate new avenues to explore the effects of miRNAs on periodontitis.

miRNAs circulate throughout the body, interacting with tissues and organs [37]. For example, hsa-let-7f-5p, hsa-miR-28-5p, hsa-miR-320d, and hsa-miR-99a-5p were found in blood in inflammatory states [38,39,40,41]. The expression of certain miRNAs may be associated with inflammation and periodontitis. Chronic periodontitis is one of the inflammatory diseases. Therefore, miRNAs are specifically expressed not only in periodontal disease, but also in other inflammatory diseases. The possibility that these miRNAs are related to systemic diseases, not to periodontal disease, cannot be ruled out. However, there was no difference between the two groups in systemic diseases. These miRNAs may be specific for periodontitis.

Moreover, miRNA expression affects mRNA and changes the expressions of other miRNAs. Periodontitis is modulated by miRNAs such as miR-181b, miR-19b, miR-23a, miR-30a, miR-let7a, miR-301a, and miR-146a [15,42]. Periodontitis may progress through complex steps involving the expression of multiple miRNAs.

In addition, miRNAs affect not only periodontal tissues, but also immune responses by cells such as monocytes and macrophages [43]. Further in vitro studies are required to elucidate the association between miRNA expression and subgingival periodontopathic bacteria in immune responses.

Several studies have also indicated relationships between salivary miRNAs and oral cancer, which suggest the use of miRNAs as a diagnostics tool of tongue squamous cell carcinoma (SCC) and biomarker of early SCC detection [44,45]. Salivary miRNAs may reflect the state of the oral mucosa. Therefore, the present findings support the increasing potential of using salivary miRNA expression as a novel predictive and diagnostic method.

In the present study, microarray analyses indicated that hsa-miR-17-3p, hsa-let-7f-5p, hsa-miR-99a-5p, hsa-miR-200a-3p, hsa-miR-28-5p, hsa-miR-320d, and hsa-miR-31-5p expressions were downregulated in the progression group compared with the control group. Conversely, expressions of these miRNAs were upregulated in RT-qPCR analyses. These results do not validate the expression directionality for the selected miRNAs, except for hsa-miR-5571-5p. One potential explanation for this discrepancy is that microarrays were performed using pooled samples for screening, whereas RT-qPCR analyses were performed using individual samples. However, RT-qPCR has higher accuracy than microarray analysis, and only high-quality RNA samples were used for analysis. Therefore, the reliability of these results was considered satisfactory. Moreover, because hsa-miR-5571-5p expression was significantly higher in the progression group than in the control group on both microarray and RT-qPCR analyses, at least the result of hsa-miR-5571-5p can be guaranteed.

There are some limitations to this study. First, the results may not be generalizable, since all patients were recruited from one hospital. Therefore, these findings must be interpreted carefully and verified in other populations. Second, the follow-up rate was 36.7%, which was low. However, there were no significant differences in parameters at baseline between the patients who were followed and not followed. The follow-up group might not be a special subgroup. Third, the observation period of this cohort study was only two years. A longer period of observation is needed in the future because the present period was less than the five years recommended by the American Academy of Periodontology and the European Federation of Periodontology [46]. Fourth, periodontitis progression was diagnosed according to CAL progression or tooth extraction [22]. The expression of unknown salivary miRNAs may be associated with other periodontal indices. Fifth, next-generation sequencing for miRNA analysis was not used because the targeted miRNA in microarray analyses are pre-specified, and the number is limited. Finally, the effects of other confounders, such as tooth-related factors, cannot be excluded [47] and warrant careful attention.

## 5. Conclusions

Based on the results of the present study, miRNAs (hsa-miR-5571-5p, hsa-let-7f-5p, hsa-miR-99a-5p, hsa-miR-28-5p, and hsa-miR-320d) could be new biomarkers for periodontitis and may help predict the progression of periodontitis. These miRNAs may contribute to early detection and precision medicine for periodontitis.

## Figures and Tables

**Table 1:** Characteristics of participants at baseline.

Variable	Category	Total (*n* = 44)	Progression (*n* = 22)	Control (*n* = 22)
Age (years)		67.1 (9.9) *	65.8 (11.1)	68.1 (8.5)
Sex	Male	15 (34.1) ^†^	8 (36.4)	7 (31.8)
	Female	29 (65.9)	14 (63.6)	15 (68.2)
Number of natural teeth present		25.3 (2.9) *	24.9 (2.5)	25.8 (3.3)
Mean PPD (mm)		2.0 (0.3) *	2.0 (0.3)	2.0 (0.3)
Mean CAL (mm)		2.3 (0.7) *	2.6 (0.8)	2.4 (0.6)
BOP (%)		7.0 (8.3) *	7.4 (9.9)	6.6 (6.3)
Plaque control record (%)		24.6 (21.8) *	20.8 (19.0)	28.4 (24.1)
Diabetes mellitus	Present	3 (6.8) ^†^	2 (9.1)	1 (4.54)

SD, standard deviation; PPD, probing pocket depth; CAL, clinical attachment level; BOP, bleeding on probing. * Mean (SD), ^†^ *n* (%).

**Table 2:** List of differentially expressed miRNAs between the progression and control groups on microarray analyses.

miRNA	Fold Change (Progression/Control)	*p*-Value *
hsa-miR-5571-5p	3.70	0.04
hsa-miR-17-3p	−6.17	<0.001
hsa-let-7f-5p	−7.90	<0.001
hsa-miR-4724-3p	−6.20	0.012
hsa-miR-99a-5p	−2.71	0.019
hsa-miR-200a-3p	−5.60	0.022
hsa-miR-28-5p	−6.86	0.029
hsa-miR-320d	−4.51	0.032
hsa-miR-31-5p	−3.83	0.045

Fold change values > 1 indicate upregulation, whereas values < 1 indicate downregulation. * Student′s *t*-test.

## Data Availability

The data used to support the findings of this study are included in the article.

## Supplementary Materials

The following are available online at https://www.mdpi.com/article/10.3390/ijerph18158010/s1, Table S1: Characteristics of non-participants at baseline; Figure S1: Ct values of U6; Table S2: Analysis of covariance with the expression of hsa-miR-5571-5p as the dependent variable; Table S3: Pathway analysis of target genes for hsa-miR-5571-5p (**A**), hsa-miR-17-3p (**B**), hsa-let-7f-5p (**C**), hsa-miR-99a-5p (**D**), hsa-miR-200a-3p (**E**), hsa-miR-28-5p (**F**), and hsa-miR-320d (**G**).
