# Supplementary material for: Detection of Salivary miRNAs That Predict Chronic Periodontitis Progression: A Cohort Study
Source: Int J Environ Res Public Health. 2021 Jul 29;18(15):8010. doi: 10.3390/ijerph18158010 (PMC8345340; doi:10.3390/ijerph18158010)
Supplement: Supplementary file 1 [file ijerph-18-08010-s001.zip › ijerph-1261194-supplementary.pdf]

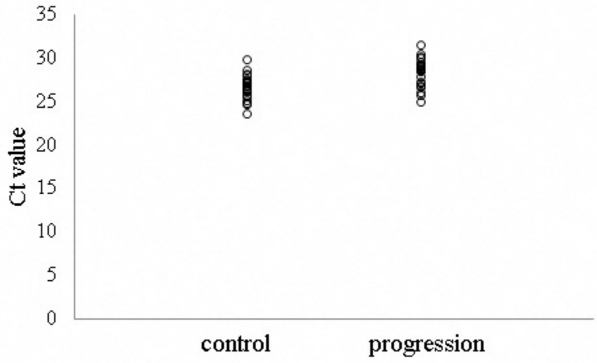

Figure S1. Ct values of U6.

Table S1. Characteristics of non-participants at baseline.

Variables	Categories	Total (n=76)
Age (years)		68.4 (9.9)*
Sex	Male	23 (30.2)†
Number of natural teeth present		23.6 (2.9)*
Mean PPD (mm)		2.0 (0.3)*
Mean CAL (mm)		2.8 (0.7)*
BOP (%)		6.9 (9.7)*
Plaque control record (%)		21.3 (20.6)*
Diabetes mellitus	Present	7 (9.2) †

* Mean (SD), † n (%).

SD, standard deviation; PPD, probing pocket depth; CAL, clinical attachment level; BOP, bleeding on probing.

Table S2. Analysis of covariance with the expression of hsa-miR-5571-5p as the dependent variable.

Variables	B (95%CI)	P value
Progression group	-0.092 (-0.162, -0.022)	0.011

The candidate variables for the model were age, gender, diabetes mellitus, PPD, BOP (%), PCR (%), and number of teeth present.

The final model was constructed based on the maximum adjusted R-squared. The F-statistic was 7.191 (p =0.011), the R-squared was 0.251, and the adjusted R-squared was 0.080. N: number. CI: confidence interval.

B: unstandardized regression coefficient. PPD: probing pocket depth. BOP: bleeding on probing. PCR: plaque control record.

Table S3. Pathway analysis of target genes for hsa-miR-5571-5p (A), hsa-miR-17-3p (B), hsa-let-7f-5p (C), hsa-miR-99a-5p (D), hsa-miR-200a-3p (E), hsa-miR-28-5p (F) and hsa-miR-320d (G).

Pathway	Target genes	*p*-value
(A)		
hsa-miR-5571-5p		
Pathways in cancer	*FGF5, GSK3B, PTCH1, MDM2, SOS1, EGLN1, MAPK1, STAT1, RARB, PIAS2, FGF2, CTNNA3, COL4A6, TGFA, PRKCB, IGF1, RUNX1T1, BMP2, GLI3, NKX3-1, ITGA2, TPM3, RALB, AKT3, FZD5, MAPK10, CRK, PIK3R3, MAPK9, SKP2, FZD4, CSF2RA, RASSF1, PIK3CD, ITGAV, FZD3, SOS2, KRAS, SMAD4, APPL1, MSH3, XIAP, TRAF3, VEGFA, DAPK2, FGF12, CBL, DVL3, CBLB, PTGS2*	9.44×10^{-10}
Insulin signaling pathway	*GSK3B, SOS1, MAPK1, FBP1, EIF4E, SHC3, PPP1R3D, G6PC, AKT3, PHKB, MAPK10, CRK, PIK3R3, SREBF1, MAPK9, PRKAB2, PRKX, PIK3CD, SOS2, KRAS, PRKAA2, PHKG1, SORBS1, PPP1R3C, CBL, CBLB, PPP1CC*	9.12×10^{-8}
ErbB signaling pathway	*GSK3B, SOS1, MAPK1, SHC3, GAB1, TGFA, PRKCB, EREG, NCK1, AKT3, MAPK10, CRK, PIK3R3, MAPK9, PIK3CD, SOS2, KRAS, CAMK2G, CBL, CBLB*	9.95×10^{-7}
Focal adhesion	*GSK3B, CAV2, SOS1, MAPK1, ACTN1, COL4A6, SHC3, PRKCB, IGF1, PPP1R12A, ITGA2, AKT3, ITGA8, PARVA, MAPK10, CRK, PIK3R3, MAPK9, ARHGAP5, PIK3CD, ITGAV, RASGRF1, SOS2, XIAP, PARVG, ITGB8, VEGFA, COL11A1, ACTN4, PPP1CC*	5.85×10^{-6}

Neurotrophin signaling pathway	*NTRK2, GSK3B, SOS1, MAPK1, IRAK4, CAMK4, MAPK13, PTPN11, SHC3, GAB1, AKT3, MAPK10, CRK, PIK3R3, MAPK9, SORT1, PIK3CD, SOS2, KRAS, YWHAB, CAMK2G, NTRK3*	1.58×10^{-5}
Tight junction	*OCLN, PARD3, ACTN1, F11R, CTNNA3, PRKCB, MAGI1, PPP2R1B, AKT3, EPB41L2, MAGI3, RAB3B, EPB41, MYH11, CLDN11, EXOC4, KRAS, INADL, AMOTL1, ACTN4, CASK, PPP2R2C*	3.06×10^{-5}
Calcium signaling pathway	*GNAL, CAMK4, CACNA1C, ADCY1, PRKCB, PPP3R2, CHRM2, PTGFR, ITPKB, SLC8A1, BST1, HRH1, HTR2C, SPECC1L, PHKB, CHRM3, PTGER3, GNAQ, SLC25A4, PRKX, HTR6, PHKG1, CAMK2G, ITPR1, DRD1, NOS1*	3.72×10^{-5}
Hepatitis C	*OCLN, GSK3B, SOS1, MAPK1, STAT1, PIAS2, MAPK13, RNASEL, PPP2R1B, AKT3, MAPK10, PIK3R3, PPARA, MAPK9, MAVS, PIK3CD, CLDN11, SOS2, KRAS, TRAF3, PPP2R2C*	1.27×10^{-4}
MAPK signaling pathway	*NTRK2, FGF5, CACNA2D1, PPM1B, RASGRP3, SOS1, MAPK1, MAP3K7, NLK, MEF2C, MAPK13, FGF2, CACNA1C, PRKCB, IL1R1, PPP3R2, RASA2, AKT3, PLA2G12A, MAPK10, TAOK1, CRK, PLA2G5, MAPK9, PRKX, MAP3K2, RASGRF1, SOS2, KRAS, RASGRF2, DUSP3, FGF12*	1.45×10^{-4}

GnRH signaling pathway	*SOS1, MAPK1, MAPK13, CACNA1C, ADCY1, PRKCB, PLA2G12A, MAPK10, PLA2G5, MAPK9, GNAQ, PRKX, MAP3K2, SOS2, KRAS, CAMK2G, ITPR1*	1.87×10^{-4}
(B)		
hsa-miR-17-3p		
Pathways in cancer	*CDK6, PPARD, CYCS, NCOA4, TRAF5, EGLN1, KITLG, DCC, FGF2, CTNNA1, ABL1, WNT9B, TGFBR1, IGF1, GLI3, COL4A5, RAC2, E2F1, ITGA2, ETS1, E2F2, CASP9, PDGFA, AKT3, BRAF, SUFU, MAPK10, TCEB1, PIK3R3, MAPK9, EGF, TRAF6, PML, FZD2, WNT10B, FZD4, WNT5A, PDGFRB, FZD3, SMAD3, ARNT2, KRAS, APPL1, TRAF3, PIK3CG, TP53, CDH1, FGF12, AKT2, CBL, STAT5A, CRKL, APC2, RALGDS, PTGS2*	1.16×10^{-11}
Colorectal cancer	*CYCS, DCC, TGFBR1, RAC2, CASP9, AKT3, BRAF, MAPK10, PIK3R3, MAPK9, SMAD3, KRAS, APPL1, PIK3CG, TP53, AKT2, APC2, RALGDS*	1.59×10^{-7}
Glioma	*CALML3, CDK6, CAMK2D, CAMK2B, SHC3, IGF1, E2F1, E2F2, PDGFA, AKT3, BRAF, PIK3R3, EGF, PDGFRB, KRAS, PIK3CG, TP53, AKT2*	1.69×10^{-7}
Neurotrophin signaling pathway	*NTRK2, CALML3, CAMK2D, CAMK2B, MAPK13, ABL1, YWHAZ, SHC3, RIPK2, TP73, AKT3, BRAF, MAPK10, PIK3R3, MAPK9, TRAF6, YWHAG, IRAK3, SORT1, KRAS, YWHAB, PIK3CG, TP53, PDK1, AKT2, CRKL*	1.76×10^{-7}
Wnt signaling pathway	*PPARD, CAMK2D, CAMK2B, WNT9B, NFATC3, PPP2R5A, RAC2, CACYBP, NFATC4, PPP2R1B, MAPK10, PPP2R5C,*	2.03×10^{-7}

	MAPK9, PPP2CA, FZD2, WNT10B, FZD4, SFRP4, WNT5A, NFATC2, PPP3CA, FZD3, NFAT5, SMAD3, NKD1, BTRC, TBL1X, TP53, APC2	
Pancreatic cancer	*CDK6, TGFBR1, RAC2, E2F1, E2F2, CASP9, AKT3, BRAF, MAPK10, PIK3R3, MAPK9, EGF, SMAD3, KRAS, PIK3CG, TP53, AKT2, RALGDS*	8.76×10^{-7}
Hepatitis C	*STAT2, MAPK13, CLDN19, PPP2R1B, AKT3, BRAF, MAPK10, PIK3R3, MAPK9, EIF2AK1, EGF, TRAF6, MAVS, PPP2CA, TRADD, KRAS, TRAF3, PIK3CG, TP53, SCARB1, PDK1, AKT2, IFNAR1, PPP2R2D, LDLR*	1.53×10^{-6}
Melanoma	*CDK6, FGF2, IGF1, E2F1, E2F2, PDGFA, AKT3, BRAF, PIK3R3, EGF, PDGFRB, KRAS, PIK3CG, TP53, CDH1, FGF12, AKT2*	3.97×10^{-6}
Chronic myeloid leukemia	*CDK6, ABL1, SHC3, TGFBR1, E2F1, E2F2, AKT3, BRAF, PIK3R3, SMAD3, KRAS, PIK3CG, TP53, AKT2, CBL, STAT5A, CRKL*	6.78×10^{-6}
Tuberculosis	*CALML3, CYCS, ATP6V0D2, CAMK2D, CAMK2B, MAPK13, NFYA, RIPK2, ARHGEF12, CASP10, TLR4, CASP9, AKT3, ATP6AP1, MYD88, MAPK10, MAPK9, HLA-DOB, VDR, TRAF6, TRADD, PPP3CA, CLEC7A, ATP6V0B, HLA-DQB1, AKT2*	4.83×10^{-5}

(C)

hsa-let-7f-5p		
Pathways in cancer	*BCL2L1, COL4A6, TGFBR1, PRKCB, IGF1, RUNX1T1, IGF1R, NRAS, STAT3, FAS, AR, RALB, SMAD2, BRAF, FASLG, FZD5, MAPK8, CDKN1A, CASP3, FZD4, FZD3, FGF11, ARNT2, ZBTB16, TP53, CDH1, DAPK2, PDGFB, AKT2, WNT1, CBL, DVL3, APC2*	3.59×10^{-9}
MAPK signaling pathway	*MRAS, MAPK11, NGF, MAP4K4, TGFBR1, PRKCB, NRAS, RASGRP1, FAS, MAP3K1, BRAF, ELK4, FASLG, MAPK8, PAK1, CASP3, CACNB4, MAP4K3, PTPN7, FGF11, PLA2G3, MAP2K7, RPS6KA3, TP53, DUSP3, PDGFB, AKT2, PLA2G2F, DUSP22*	4.05×10^{-9}
p53 signaling pathway	*CCNG2, IGF1, SESN3, CHEK1, FAS, CDKN1A, CASP3, STEAP3, MDM4, TP53, RRM2*	6.24×10^{-5}
Cytokine-cytokine receptor interaction	*TGFBR1, CCL15, OSM, CCL7, FAS, EDA, PRLR, FASLG, CCL3, IL13, TNFRSF1B, CCL16, OSMR, CCR7, TNFSF9, TNFSF10, IL22RA1, PDGFB, IL17RA, CCL22*	4.08×10^{-4}
Apoptosis	*NGF, BCL2L1, FAS, PRKAR2A, FASLG, AIFM1, CASP3, CFLAR, TP53, TNFSF10, AKT2*	4.59×10^{-4}
Melanoma	*IGF1, IGF1R, NRAS, BRAF, CDKN1A, FGF11, TP53, CDH1, PDGFB, AKT2*	5.29×10^{-4}
Glioma	*PRKCB, IGF1, IGF1R, NRAS, BRAF, CDKN1A, TP53, PDGFB, AKT2*	8.31×10^{-4}

Prostate cancer	*CREB3L2, IGF1, IGF1R, NRAS, AR, BRAF, CDKN1A, TP53, PDGFB, AKT2*	$1.14×10^{-3}$
Neurotrophin signaling pathway	*MAPK11, NGF, NRAS, MAP3K1, YWHAE, BRAF, FASLG, MAPK8, MAP2K7, RPS6KA3, TP53, AKT2*	$1.21×10^{-3}$
Adherens junction	*PTPRB, ACP1, TGFBR1, MLLT4, IGF1R, SMAD2, INSR, VCL, CDH1*	$1.27×10^{-3}$
(D)		
hsa-miR-99a-5p		
Chemokine signaling pathway	*CXCL16, RAP1B, FOXO3, STAT5B*	$1.99×10^{-2}$
(E)		
hsa-miR-200a-3p		
Neurotrophin signaling pathway	*NTRK2, MAPK1, IRAK4, CAMK4, IRS1, ABL1, PTPN11, IRS4, SHC3, GAB1, TP73, RPS6KA6, MAP3K3, YWHAE, BRAF, MAPK10, CRK, FRS2, PIK3R3, MAPK9, MAPK8, TRAF6, YWHAG, KIDINS220, KRAS, MAP2K7, RPS6KA3, TP53, FOXO3, IRS2, PSEN1, SH2B3*	$2.74×10^{-12}$
MAPK signaling pathway	*NTRK2, CACNA2D1, MAPK1, MAP4K4, MEF2C, CACNA1E, PRKCB, TGFBR2, RASA2, PPM1A, IL1A, RPS6KA6, MAP3K3, PDGFRA, LAMTOR3, PDGFA, DUSP6, MAP2K4, BRAF, CACNA1B, ELK4, MAPK10, TAOK1, PRKCA, CRK, MAPK9, MAPK8, CDC25B, FGFR1, TGFB2, TRAF6, PAK1, EGFR, GNG12, MAP3K2, PDGFRB,*	$4.01×10^{-12}$

	TAB2, KRAS, RASA1, RASGRF2, MAP2K7, RPS6KA3, ARRB1, TP53, DUSP3, NF1, STK3, PRKACB	
Insulin signaling pathway	*EXOC7, MAPK1, IRS1, IRS4, SHC3, PDE3A, PRKAR2B, PRKAR1A, G6PC, INSR, BRAF, PRKAR2A, RHEB, MAPK10, CRK, PIK3R3, MAPK9, PDE3B, MAPK8, TSC1, PHKA1, PCK1, KRAS, PRKAA2, PRKCI, PRKAB1, PDPK1, PHKG2, PPARGC1A, CBL, PRKACB, IRS2*	1.35×10^{-11}
Wnt signaling pathway	*SIAH1, PLCB1, AXIN1, WNT16, PRKCB, CSNK1E, CCND2, VANGL1, SMAD2, PLCB4, PPP2R1B, PLCB2, MAPK10, PRKCA, PPP2R5C, MAPK9, PPP2R5E, MAPK8, CTBP2, PPP2CA, WNT8A, FZD8, WNT5A, FZD3, NKD1, TBL1XR1, SMAD4, TP53, NFATC1, SOX17, FBXW11, PRKACB, APC2, PSEN1*	1.61×10^{-11}
Protein processing in the endoplasmic reticulum	*SEC63, BAG2, UFD1L, SEC61A1, CANX, YOD1, SEC23A, PARK2, DNAJC5, ATF6, BAG1, UBE2G1, ERN1, HSPA4L, MAPK10, SEC24A, ATXN3, MAPK9, SAR1A, MAN1C1, DDOST, MAPK8, WFS1, EDEM1, UBE2D4, SEL1L, MAP2K7, RAD23B, MBTPS2, SSR1, LMAN1*	8.45×10^{-9}
Endocytosis	*USP8, RAB22A, EHD3, ADRB1, DNM1, IGF1R, TGFBR2, GRK6, EHD1, PIP5K1A, SMAD2, PDGFRA, GIT2, SRC, CXCR1, TGFB2, TRAF6, PARD6B, EGFR, FLT1, TFRC, RAB31, RAB5A, AP2B1, VPS37A, PRKCI, RET, ARAP2, ADRBK2, ARRB1, EPN2, CBL, RAB11FIP4, RNF41*	1.50×10^{-8}

ErbB signaling pathway	*MAPK1, ABL1, SHC3, GAB1, PRKCB, ABL2, MAP2K4, BRAF, MAPK10, PRKCA, CRK, PIK3R3, MAPK9, SRC, MAPK8, PAK1, EGFR, KRAS, MAP2K7, STAT5B, CBL, STAT5A*	1.68×10^{-8}
Chronic myeloid leukemia	*MAPK1, ABL1, PTPN11, SHC3, E2F3, TGFBR2, RUNX1, GAB2, BRAF, CRK, PIK3R3, TGFB2, CTBP2, KRAS, SMAD4, TP53, STAT5B, CBL, STAT5A*	8.60×10^{-8}
Focal adhesion	*MAPK1, ITGA6, TLN2, SHC3, PRKCB, PDGFD, IGF1R, CCND2, PTEN, PDGFRA, THBS1, PDGFA, BRAF, MAPK10, PRKCA, CRK, PIK3R3, VCL, MAPK9, SRC, MAPK8, PAK1, EGFR, FLT1, MYLK3, ITGAV, PDGFRB, LAMC2, XIAP, ITGB8, PDPK1, HGF, ITGA11*	8.91×10^{-8}
(F)		
hsa-miR-28-5p		
Neurotrophin signaling pathway	*NTRK2, IKBKB, GSK3B, MAPK1, YWHAH, CAMK2D, YWHAZ, PTPN11, NRAS, RAP1A, TP73, MAP3K3, RAP1B, BRAF, SH2B1, FRS2, IRAK3, SORT1, NGFR, CAMK2G, RPS6KA1, AKT2, RAPGEF1, NTRK3, SH2B3*	7.80×10^{-9}
Axon guidance	*GSK3B, GNAI1, MAPK1, DCC, SEMA5A, SEMA4B, DPYSL2, SEMA7A, PPP3R2, NRAS, EFNB3, ABLIM3, EFNB1, PLXNA1, ABLIM1, SEMA4G, PLXNA2, NFATC2, NFAT5, NTN1, SEMA4F, EPHA5, CXCL12, PAK2, LIMK2*	8.04×10^{-9}
MAPK signaling pathway	*NTRK2, PTPRR, IKBKB, CACNG2, MAPK1, MAP3K7, CACNA1C, CACNA1E, MAP3K13, DUSP16, PPP3R2,*	6.69×10^{-8}

	NRAS, GNA12, RAP1A, DUSP4, MAP3K3, CACNG8, CACNA1I, RAP1B, BRAF, PRKCA, PLA2G5, PPP5C, GNG12, SRF, MAP3K2, NFATC2, FGF11, FGF23, ARRB1, RPS6KA1, PAK2, CACNG5, AKT2, STK4	
Long-term potentiation	*MAPK1, CAMK2D, CACNA1C, ADCY1, PPP3R2, NRAS, RAP1A, GRM5, RAP1B, BRAF, PRKCA, GRIN2B, RAPGEF3, GRM1, PPP1R1A, CAMK2G, RPS6KA1*	7.91×10^{-8}
Glutamatergic synapse	*ADCY2, GNAI1, MAPK1, CACNA1C, ADCY1, PPP3R2, PLD2, GRM5, PRKCA, GRIN2B, PLA2G5, GRIK3, GRM1, SLC1A2, GNG12, GNG2, GRIA4, SHANK2, GRIN3A, SLC38A2, GRM4, SLC1A7*	2.85×10^{-7}
Calcium signaling pathway	*SLC8A2, AVPR1A, ADCY2, CAMK2D, CACNA1C, CACNA1E, ADCY1, CD38, PPP3R2, ITPKB, BST1, HRH2, GRM5, CACNA1I, PHKB, PRKCA, GRM1, PTGER3, P2RX5, P2RX4, P2RX1, PLCD3, CAMK2G, PHKG2, NOS1, PDE1A*	5.12×10^{-7}
Pathways in cancer	*WNT8B, IKBKB, GSK3B, PTCH1, MAPK1, PIAS2, DCC, CTNNA3, PGF, CCDC6, NRAS, PTEN, BCR, BRAF, SUFU, TCF7, PRKCA, FADD, FZD1, CDKN1A, WNT5B, VHL, FZD8, FGF11, FGF23, ARNT2, ZBTB16, PAX8, TRAF1, AKT2, STAT5B, STK36, CBL, PTGS2, STK4*	6.66×10^{-6}
Melanogenesis	*WNT8B, GSK3B, ADCY2, GNAI1, MAPK1, CAMK2D, ADCY1, CREB3L2, NRAS, CREB1, TCF7, PRKCA, FZD1, WNT5B, FZD8, CAMK2G*	6.21×10^{-5}

Cell adhesion molecules	*SDC1, SDC4, GLG1, SIGLEC1, NFASC, CLDN19, CD276, PDCD1LG2, SDC3, NCAM1, CD34, PDCD1, PVR, PVRL3, SELL, CD274, CD28, PTPRF*	8.57×10^{-5}
Neuroactive ligand-receptor interaction	*AVPR1A, P2RY2, P2RY8, HRH4, HRH2, GRM5, VIPR1, ADCYAP1R1, PRLR, GRIN2B, GPR83, GABRA3, GRIK3, GRM1, PTGER3, P2RX5, FSHB, P2RX4, P2RX1, GRIA4, NPY2R, AVPR2, GRIN3A, HCRTR1, CHRNA4, THRB, GRM4, CHRNB2*	1.38×10^{-5}
(G)		
hsa-miR-320d		
Pathways in cancer	*VEGFB, CDK6, FGF5, PPARD, IKBKB, PTCH1, CYCS, MDM2, COL4A4, CDC42, FGF9, TCF7L1, MET, LEF1, MAPK1, WNT11, STAT1, MAPK3, LAMA3, AXIN2, KITLG, WNT4, PIAS2, DCC, CUL2, FGF1, WNT7B, HIF1A, FGF2, CTNNA1, CTNNA3, PGF, COL4A6, FZD6, ARNT, ABL1, KIT, WNT9B, BIRC5, TGFA, E2F3, FOXO1, WNT2B, MAX, EPAS1, AXIN1, TGFBR1, PRKCB, IGF1, PIK3CA, PIK3CB, RUNX1T1, FZD7, BMP2, MMP2, GLI3, IGF1R, PRKCG, NKX3-1, TGFBR2, NRAS, COL4A5, CDK4, RAC2, PIK3R2, STAT3, E2F1, CCND1, PTEN, ITGA2, RAD51, ETS1, TPM3, AR, GRB2, E2F2, SMAD2, PDGFRA, FGF17, FGF19, CASP9, PDGFA, AKT3, RUNX1, RASSF5, BRAF, SUFU, PIAS3, CSF1R, TCF7, HHIP, FZD5, MAPK10, TCEB1, PRKCA, CRK, PIK3R3, FADD, TPR, MAPK9, LAMC1,*	1.02×10^{-38}

	MAPK8, FGFR1, TGFB2, SKP2, CDKN1A, TRAF6, PML, PTCH2, CTBP2, FZD9, PLCG1, LAMA4, EGFR, WNT8A, CTNNA2, BID, CASP3, PIK3R1, WNT10B, FZD4, LAMA1, RAF1, JUN, VHL, FZD8, CSF2RA, MITF, CKS1B, PIK3CD, ITGAV, WNT5A, PDGFRB, BCL2, LAMC2, RAC1, FZD3, PTK2, BAX, SMAD3, ITGB1, FGF11, TCF7L2, WNT3, SOS2, FGF23, ARNT2, KRAS, SMAD4, ZBTB16, APPL1, RET, RB1, RELA, MSH3, XIAP, TRAF3, CEBPA, SLC2A1, PIK3CG, CTNNB1, GLI2, CDKN2A, RARA, RALA, TP53, RBX1, PAX8, CDH1, TRAF1, HGF, VEGFA, RXRA, DAPK2, FGF12, PDGFB, HDAC2, AKT2, STAT5B, CBL, CRKL, FN1, APC2, RALGDS, FGFR2, STK4	
Neurotrophin signaling pathway	*NTRK2, CALML3, IKBKB, CDC42, MAPK1, RPS6KA5, YWHAH, SHC4, CAMK2D, MAPK3, CAMK2B, IRAK4, CAMK4, IRS1, MAPK13, PRDM4, ABL1, YWHAZ, PTPN11, SHC3, PIK3CA, PIK3CB, YWHAQ, BDNF, RIPK2, NRAS, PIK3R2, MAPKAPK2, RAP1A, TP73, GRB2, RPS6KA6, MAP3K1, MAP3K3, IRAK2, YWHAE, AKT3, BRAF, MAPK10, CRK, FRS2, PIK3R3, MAPK9, CAMK2A, MAPK8, IRAK1, SHC2, TRAF6, RPS6KA2, MAPK7, YWHAG, PLCG1, PIK3R1, RAF1, IRAK3, MAPK12, NFKBIE, SORT1, JUN, PIK3CD, MAPK14, NGFR, BCL2, RAC1, BAX, NTF3, SOS2, KRAS, RELA, MAP2K7, RPS6KA3, CAMK2G, PIK3CG, TP53, RPS6KA1, FOXO3,*	4.15×10^{-25}

	PDK1, CALM3, AKT2, RAPGEF1, CRKL, NTF4, NTRK3, PSEN1, SH2B3	
Focal adhesion	*VEGFB, FLT4, CAV2, ITGA1, COL4A4, CDC42, MET, MAPK1, SHC4, MAPK3, LAMA3, PGF, COL4A6, TNR, CHAD, TLN2, SHC3, ITGA4, PRKCB, MYLK, IGF1, COL5A3, PIK3CA, PIK3CB, PPP1R12A, ROCK2, PDGFD, IGF1R, PRKCG, CCND2, BCAR1, COL4A5, RAC2, PIK3R2, DIAPH1, CCND1, PTEN, ITGA2, RAP1A, MYL12B, GRB2, PAK6, PDGFRA, TLN1, FLNC, PDGFA, AKT3, BRAF, MYLK2, PARVA, MAPK10, ITGA5, PRKCA, CRK, PIK3R3, TNXB, MAPK9, SRC, LAMC1, CAPN2, CAV1, ARHGAP5, MAPK8, SHC2, ITGA10, PAK1, LAMA4, EGFR, FLT1, VAV2, PIK3R1, LAMA1, RAF1, JUN, MYLK3, THBS2, ACTN2, COL6A2, PIK3CD, ITGAV, TNC, PDGFRB, BCL2, LAMC2, RAC1, PTK2, ITGB1, RASGRF1, SOS2, PAK3, COL5A1, XIAP, PAK7, CCND3, PARVG, PIK3CG, CTNNB1, MYL12A, ITGB8, MYL5, PDPK1, HGF, PAK2, VEGFA, ITGB5, PDGFB, ACTN4, ITGA11, AKT2, RAPGEF1, CRKL, FN1, PPP1CB, VAV3*	2.98×10^{-24}
MAPK signaling pathway	*NTRK2, FGF5, MRAS, PPM1B, RASGRP3, IKBKB, CACNG2, CDC42, FGF9, MAPK1, RPS6KA5, MAP3K7, MAPK3, RASGRP4, MAP3K14, FGF1, MAPK13, FGF2, CACNA1C, CACNA1E, MAX, MAP3K13, TGFBR1, PRKCB,*	1.38×10^{-23}

	BDNF, IL1R1, DUSP16, PPP3R2, MAPT, PRKCG, TGFBR2, NRAS, RAC2, RASGRP1, CACNA1D, MAPKAPK2, TNFRSF1A, GNA12, CACNG4, RAP1A, CACNB1, GRB2, DUSP4, RASA2, PPM1A, RPS6KA6, MAP3K1, TAB1, MAP3K3, PDGFRA, FGF17, NFATC4, FGF19, CACNG8, FLNC, CACNA2D2, MAP3K12, PDGFA, PRKACA, AKT3, CACNA1I, BRAF, CACNA1B, PLA2G6, ELK4, MAPK10, TAOK1, PRKCA, CRK, PLA2G5, PPP5C, MAPK9, MAPK8, PPP3R1, FGFR1, TGFB2, MAP3K11, TRAF6, RPS6KA2, PAK1, MAPK7, EGFR, GNG12, CASP3, CACNB4, RAF1, MAPK12, JUN, SRF, PRKX, MAP3K2, MKNK1, MAPK14, RRAS2, CACNG7, PDGFRB, NFATC2, TAB2, RAC1, MAPK8IP3, NTF3, FGF11, ATF2, RASGRF1, SOS2, FGF23, TAOK3, TAOK2, KRAS, RASA1, RASGRF2, PLA2G3, RELA, MAP2K7, RPS6KA3, PPP3CB, ARRB1, TP53, CACNB3, RPS6KA1, DUSP3, PAK2, NF1, FGF12, PDGFB, AKT2, STK3, CRKL, PLA2G2F, DUSP14, CACNB2, NTF4, PRKACB, PLA2G4E, DUSP22, FGFR2, STK4	
Endocytosis	*STAMBP, CAV2, MDM2, CXCR4, CDC42, RAB22A, RAB11B, MET, ADRB2, RAB11FIP5, EHD3, SH3KBP1, ASAP1, RAB7A, PARD3, KIT, TGFBR1, VPS36, ADRBK1, F2R, DNAJC6, RAB4A, IGF1R, RAB11FIP3, TGFBR2, VPS4B, GRK6, AP2S1, RAB5B, SH3GLB1, RAB11A, DNM2, IQSEC1, CHMP5, EHD1, PIP5K1A, ASAP2, ITCH, CXCR2,*	$1.63{\times}10^{-23}$

	SMAD2, PDGFRA, EHD2, ERBB4, CHMP1B, NEDD4L, AP2A2, NEDD4, RAB11FIP1, CSF1R, CHMP6, DNM3, SH3GL1, GIT2, DNM1L, CHMP4C, SMURF2, SRC, AGAP2, CAV1, TGFB2, TRAF6, PML, EHD4, STAM2, PARD6B, EGFR, PSD3, FLT1, ADRB3, CHMP2B, TFRC, SH3GL2, IQSEC3, IL2RA, PSD4, CLTCL1, RAB31, PRKCZ, CCR5, ZFYVE16, AP2B1, SMAD3, IQSEC2, SMAP2, PRKCI, RET, VPS37D, ZFYVE20, ADRBK2, DAB2, ARRB1, EEA1, PDCD6IP, AGAP1, EPS15, RAB11FIP2, ACAP2, IL2RB, EPN2, ZFYVE9, CBL, RAB11FIP4, SMURF1, RNF41, LDLRAP1, ARF6, VPS45, VPS37B, PARD6G, SMAP1, FGFR2	
Calcium signaling pathway	*SLC8A2, AVPR1A, CALML3, ATP2B1, GNA14, RYR3, ADCY2, GNAL, ADRB2, CAMK2D, CAMK2B, ATP2A2, CAMK4, ATP2B4, HTR2A, CACNA1C, BDKRB2, CACNA1E, PTK2B, ADCY1, VDAC1, EDNRB, PRKCB, MYLK, RYR2, F2R, CYSLTR1, PPP3R2, PRKCG, ADCY9, DRD5, BDKRB1, CACNA1D, CHRM2, ITPKB, PPID, SLC8A1, CYSLTR2, BST1, HTR5A, HRH2, PDGFRA, PHKA2, HRH1, CHRNA7, ERBB4, PRKACA, HTR2C, SPECC1L, CACNA1I, PLCB2, MYLK2, PHKB, CACNA1B, EDNRA, OXTR, PRKCA, ITPKA, ADCY3, P2RX7, ATP2A3, CAMK2A, AGTR1, GRM1, PPP3R1, HTR7, ITPR2, PTGER3, ADRA1B, SPHK1, ATP2B2, PLCB3, P2RX5,*	1.71×10^{-23}

	GNAQ, SLC25A4, PLCG1, EGFR, ADRB3, HTR4, PHKA1, PTAFR, P2RX2, P2RX1, MYLK3, PRKX, PDGFRB, SLC8A3, GRIN1, GRIN2A, HTR6, PLCD3, PHKG1, CAMK2G, PPP3CB, RYR1, PDE1B, PHKG2, NOS1, CALM3, PDE1A, PRKACB, ADRA1D, TACR1, ATP2B3	
Axon guidance	*SLIT3, EPHA8, NGEF, SEMA3C, CXCR4, CDC42, GNAI1, MET, MAPK1, MAPK3, UNC5A, DCC, LIMK1, SRGAP1, SEMA5A, ABL1, DPYSL2, SEMA7A, NFATC3, PPP3R2, SEMA6D, ROCK2, L1CAM, NRAS, CFL2, ARHGEF12, EFNB3, RAC2, SEMA3G, PLXNB3, EPHB1, PAK6, ABLIM2, EFNB1, SEMA6A, NFATC4, NCK1, ROBO2, EPHA7, PLXNA1, GNAI3, EFNA5, NTN4, ABLIM1, PPP3R1, NCK2, UNC5B, PLXNB1, PAK1, SRGAP3, PLXNA2, SEMA3D, SEMA3E, GNAI2, PLXNC1, NFATC2, SEMA3F, RAC1, RGS3, NRP1, PTK2, NFAT5, ITGB1, PAK3, KRAS, RASA1, SEMA4F, PAK7, SEMA3A, EPHA5, PPP3CB, CXCL12, EPHA4, PLXNB2, EPHB4, PAK2, DPYSL5, SRGAP2, UNC5C, LIMK2*	3.31×10^{-20}
Glioma	*CALML3, CDK6, MDM2, MAPK1, SHC4, CAMK2D, MAPK3, CAMK2B, SHC3, TGFA, E2F3, PRKCB, IGF1, PIK3CA, PIK3CB, IGF1R, PRKCG, NRAS, CDK4, PIK3R2, E2F1, CCND1, PTEN, GRB2, E2F2, PDGFRA, PDGFA, AKT3, BRAF, PRKCA, PIK3R3, CAMK2A, SHC2, CDKN1A, PLCG1, EGFR, PIK3R1, RAF1, PIK3CD, PDGFRB, SOS2,*	9.96×10^{-20}

	KRAS, RB1, CAMK2G, PIK3CG, CDKN2A, TP53, PDGFB, CALM3, AKT2	
Regulation of the actin cytoskeleton	*ARHGEF7, FGF5, MRAS, CHRM4, SLC9A1, ARHGEF6, ITGA1, ARPC5, CDC42, FGF9, MAPK1, ARPC2, MAPK3, FGF1, LIMK1, ARHGEF1, FGF2, TIAM1, BDKRB2, ENAH, ITGA4, ARPC1B, MYLK, ABI2, PIK3CA, PIK3CB, F2R, PPP1R12A, ROCK2, PDGFD, NRAS, BCAR1, ITGAM, MYH10, CFL2, ARHGEF12, RAC2, BDKRB1, PIK3R2, CHRM2, DIAPH1, GNA12, ITGA2, MSN, MYL12B, PIP5K1A, PAK6, SCIN, PDGFRA, FGF17, FGF19, PIP4K2B, PDGFA, BRAF, MYLK2, GNA13, ITGA5, CRK, PIK3R3, PFN1, FGFR1, ITGA10, PAK1, EGFR, GNG12, PIKFYVE, VAV2, PIK3R1, RAF1, ITGAL, MYLK3, ACTN2, PIK3CD, ITGAV, RRAS2, PDGFRB, IQGAP1, RAC1, PTK2, ITGB1, FGF11, WASF2, SOS2, FGF23, PAK3, FGD1, ARHGEF4, KRAS, BAIAP2, PAK7, PIK3CG, CYFIP1, MYL12A, ITGB8, MYL5, PAK2, ITGB5, CYFIP2, FGF12, PDGFB, ACTN4, ITGA11, RDX, CRKL, FN1, APC2, PPP1CB, FGFR2, VAV3, LIMK2*	$2.20{\times}10^{-19}$
